# Fertility Trends and Adverse Pregnancy Outcomes in Female Patients With Psoriasis in the UK

**DOI:** 10.1001/jamadermatol.2023.1400

**Published:** 2023-06-07

**Authors:** Teng-Chou Chen, Ireny Y. K. Iskandar, Rosa Parisi, Matthias Pierce, Clare Tower, C. Elise Kleyn, Christopher E. M. Griffiths, Darren M. Ashcroft

**Affiliations:** 1 Centre for Pharmacoepidemiology and Drug Safety, Division of Pharmacy and Optometry, School of Health Sciences, Faculty of Biology, Medicine and Health, The University of Manchester, Manchester, England, United Kingdom; 2 Centre for Occupational and Environmental Health, Division of Population Health, Health Services Research and Primary Care, School of Health Sciences, Faculty of Biology, Medicine and Health, The University of Manchester, Manchester, England, United Kingdom; 3 Division of Informatics, Imaging and Data Sciences, Manchester Academic Health Sciences Centre, School of Health Sciences, Faculty of Biology, Medicine and Health, The University of Manchester, Manchester, England, United Kingdom; 4 Centre for Women’s Mental Health, Division of Psychology and Mental Health, School of Health Sciences, Faculty of Biology, Medicine and Health, The University of Manchester, Manchester, England, United Kingdom; 5 Division of Obstetrics, St Mary’s Hospital, Manchester University NHS Foundation Trust, Manchester Academic Health Science Centre, Manchester, England, United Kingdom; 6 Centre for Dermatology Research, Salford Royal NHS Foundation Trust, The University of Manchester, Manchester Academic Health Science Centre, National Institute for Health and Care Research (NIHR) Manchester Biomedical Research Centre, Manchester, England, United Kingdom; 7 National Institute for Health and Care Research (NIHR) Greater Manchester Patient Safety Translational Research Centre, Manchester Academic Health Science Centre, The University of Manchester, Manchester, England, United Kingdom

## Abstract

**Importance:**

Evidence regarding fertility trends and obstetric outcomes among patients with psoriasis is limited by studies of small sample sizes, noninclusion of comparators, and the lack of accurate pregnancy records.

**Objective:**

To investigate fertility rates and obstetric outcomes of pregnancies in female patients with psoriasis compared with age- and general practice–matched comparators without psoriasis.

**Design, Setting, and Participants:**

This population-based cohort study used data from 887 primary care practices that contributed to the UK Clinical Practice Research Datalink GOLD database between 1998 and 2019, linked to a pregnancy register and Hospital Episode Statistics. There were 6 223 298 patients of common childbearing ages (15-44 years), and 63 681 patients with psoriasis had at least 1 year of follow-up data prior to the diagnosis of psoriasis. For each patient with psoriasis, 5 patients were matched by age from the same general practice. The median follow-up duration was 4.1 years. Data analysis was performed in 2021.

**Exposures:**

Patients with psoriasis were identified using clinical diagnostic codes from consultations.

**Main Outcomes and Measures:**

Fertility rates were calculated as the number of pregnancies per 100 patient-years. The outcomes of each pregnancy recorded in the pregnancy register or Hospital Episode Statistics were screened to identify obstetric outcomes. A negative binomial model was used to examine the association between psoriasis and the fertility rate. Logistic regression was applied to compare the association between psoriasis and obstetric outcomes.

**Results:**

A total of 63 681 patients with psoriasis and 318 405 matched comparators were included in the analysis (median [IQR] age, 30 [22-37] years). Lower fertility rates (rate ratio, 0.75; 95% CI, 0.69-0.83) were found in patients with moderate to severe psoriasis. Compared with matched comparators without psoriasis, pregnancies in patients with psoriasis had a higher risk of loss (odds ratio, 1.06; 95% CI, 1.03-1.10); however, there was no increase in the risks of antenatal hemorrhage, preeclampsia, or gestational diabetes.

**Conclusion and Relevance:**

In this cohort study, patients with moderate to severe psoriasis had a lower fertility rate, and the risk of pregnancy loss was higher than in matched comparators without psoriasis. Future research should identify the mechanism of increased risk of pregnancy loss among patients with psoriasis.

## Key Points

**Question:**

What are the fertility rates and birth outcomes in female patients with psoriasis when compared with age- and practice-matched patients without psoriasis?

**Findings:**

This cohort study included 63 681 patients with psoriasis and 318 405 matched comparators of common reproductive ages from the UK Clinical Practice Research Datalink from 1998 to 2019. Patients with moderate to severe psoriasis had lower fertility rates, and there was a slightly higher risk of pregnancy loss in patients with psoriasis.

**Meaning:**

Because of the increased risk of pregnancy loss in patients with psoriasis, particularly occurring during the first trimester, future research should identify the mechanism of pregnancy loss in patients with psoriasis.

## Teaser

This cohort study investigates fertility rates and obstetric outcomes of pregnancies in female patients with psoriasis compared with age- and general practice–matched comparators without psoriasis.

## Introduction

Psoriasis is an immune-mediated, inflammatory skin condition that has been recognized by the World Health Organization as a noncommunicable disease.^1^ Half of patients with psoriasis are women in whom the age of diagnosis is often before 40 years.^2,3^ When coinciding with the peak reproductive period, a diagnosis of psoriasis could affect childbearing potential.

Inflammatory and autoimmune diseases have been associated with negative pregnancy outcomes.^4^ It has been hypothesized that proinflammatory cytokines affect endothelial cells and result in systemic and placental vasculopathy.^4^ Psoriasis and its management could lead to a lower fertility rate and negative pregnancy outcomes, such as miscarriage, for several reasons. First, psoriasis can cause an alteration in body image and is often associated with low self-esteem and stigmatization, and may adversely affect social functioning.^5,6^ Patients with moderate to severe psoriasis may be treated with systemic therapies and need appropriate counselling regarding contraception.^7,8^ Moreover, unhealthy lifestyle behaviors, such as smoking, and multimorbidity, such as depression and metabolic syndrome, are more common among patients with psoriasis and are also linked to adverse pregnancy outcomes.^9,10,11,12,13^

Studies that have examined fertility and pregnancy outcomes in women with psoriasis have reported conflicting findings.^14,15^ Most studies had small sample sizes (<100 women) and are thus likely underpowered to detect a difference in pregnancy outcomes.^14,15,16^ The majority of those studies used disease registry data or lacked a matched comparison group and hence were unable to estimate the association of fertility and adverse pregnancy outcomes in women with psoriasis when compared with the general population.^14,15,16,17^

Therefore, in this cohort study we aimed to investigate fertility and obstetric outcomes in patients with psoriasis when compared with the age- and practice-matched population using population-based electronic health records (EHRs). The objectives were to (1) explore the annual trends in fertility rates in female patients with psoriasis, (2) investigate the association between psoriasis and birth outcomes when compared with matched comparators without psoriasis, and (3) identify the association between psoriasis and adverse pregnancy outcomes.

## Methods

### Study Design and Data Sources

This population-based cohort study used EHR data from a large UK primary care database, the Clinical Practice Research Datalink (CPRD) GOLD, from 1998 to 2019, linking to the pregnancy register, Hospital Episode Statistics (HES), and an area-level measure of deprivation.^18^ The pregnancy register was generated by using Read codes to screen any primary care record in CPRD GOLD; this approach has been validated using linked HES data. The study protocol was approved by the Independent Scientific Advisory Committee of the Medicines and Healthcare Products Regulatory Agency, which waived need for patient informed consent owing to use of deidentified data. The study was reported according to the Strengthening the Reporting of Observational Studies in Epidemiology (STROBE) reporting guideline.^19^

### Study Population for Different Analytical Purposes

We identified all female patients aged 15 to 44 years during January 1998 through December 2019 to include patients of common reproductive ages. Patients with psoriasis were identified by screening relevant Read codes from clinical consultations in the EHRs. We followed patients from the latest date of receiving a diagnosis of psoriasis, age 15 years or January 1, 1998 (index date). For each patient with psoriasis, 5 comparators without psoriasis from the same primary care practice were selected and matched on year of birth. Matched comparators were assigned the same index date as the patient with psoriasis. Both patients with psoriasis and matched comparators were followed from the index date to (1) age 45 years, (2) date of death, (3) transfer out of practice, (4) last date of data collection, or (5) end of the study period (December 31, 2019), whichever occurred first. Patients were excluded from the study if they did not have at least 1 year of registration records before the index date.

**Figure 1.**
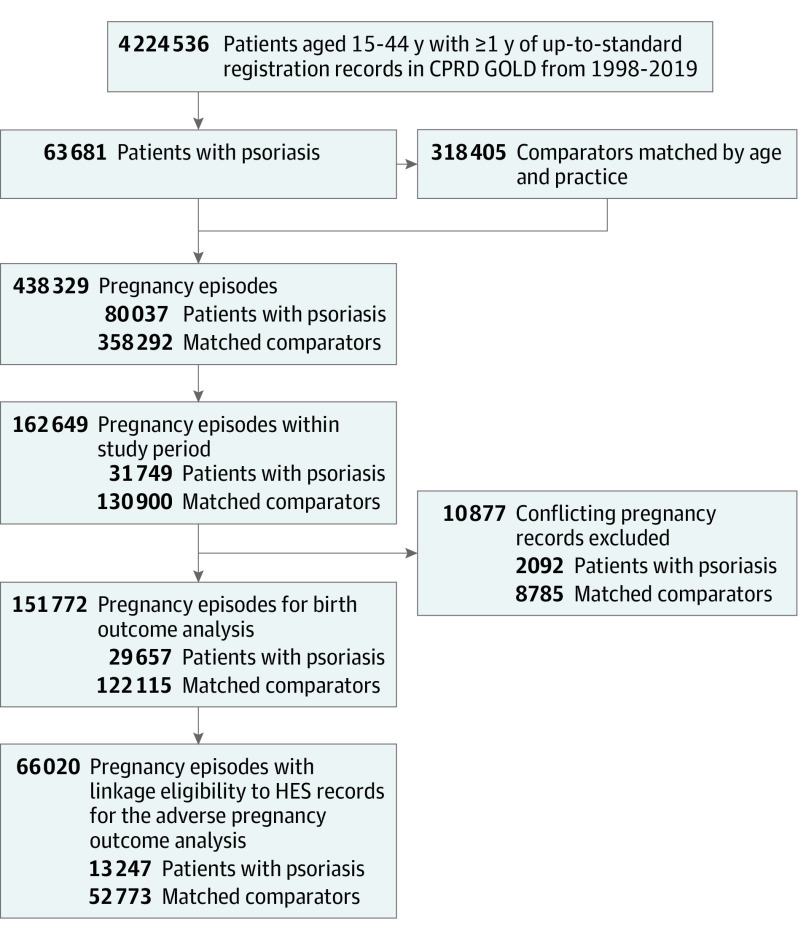
Flowchart for Selection of Female Patients With Psoriasis and Matched Comparators and Their Pregnancy Episodes CPRD indicates Clinical Practice Research Datalink; HES, Hospital Episode Statistics.

All pregnancy records from patients with psoriasis and matched comparators were extracted. Pregnancy records resulting in more than 1 live-born infant (ie, multiple pregnancy, such as twins or triplets) were excluded because there was increasing risk of severe maternal outcomes.^20^ Based on published methods of generating pregnancy registers, conflicting pregnancy records were also excluded.^18^ Pregnancy records with linkage eligibility to HES were selected to identify the association between psoriasis and adverse pregnancy outcomes (Figure 1).

### Outcome Measures

Any pregnancy episode with a start date within the follow-up period was counted to calculate fertility rate, which also included pregnancies with an outcome of stillbirth. We screened the pregnancy outcomes in the pregnancy register to categorize birth outcomes into pregnancy loss, live birth, stillbirth, and preterm birth. Pregnancy loss was defined as pregnancies recorded as miscarriage, ectopic, molar, blighted ovum, and any unspecified loss. Live births were defined as any pregnancy episode with the outcome recorded as a live birth or delivery based on a third-trimester pregnancy record or a late pregnancy record. Pregnancies with the outcome recorded as stillbirth were defined as stillbirth. Any pregnancy episode that was not recorded as pregnancy loss or termination was categorized as delivery, and delivered pregnancy episodes with a gestation period less than 37 weeks were treated as preterm birth (eTable 1 in Supplement 1).

For each pregnancy record, adverse pregnancy outcomes were identified using codes from the *International Statistical Classification of Diseases and Related Health Problems, Tenth Revision*, from HES records or Read codes from CPRD GOLD clinical files. Adverse pregnancy outcomes included venous thromboembolism, antenatal hemorrhage, preeclampsia, gestational hypertension, gestational diabetes, and cesarean delivery. Cesarean delivery was identified based on the method of delivery in the HES maternity information. Pregnancy episodes in patients with diagnosis records of hypertension or diabetes in the CPRD GOLD before the start of pregnancy were not eligible for the analysis of gestational hypertension or diabetes (eTable 1 in Supplement 1).

### Severity of Psoriasis

Patients with psoriasis were categorized as having moderate to severe psoriasis when systemic treatments (methotrexate, apremilast, acitretin, etretinate, ciclosporin, fumaric acid esters, or hydroxycarbamide), phototherapy, or biologic therapies (adalimumab, brodalumab, certolizumab, efalizumab, etanercept, guselkumab, infliximab, ixekizumab, secukinumab, tildrakizumab, and ustekinumab) were prescribed. The severity of psoriasis was a time-dependent variable, and hence the date when patients were designated with moderate to severe psoriasis was also assigned to their matched comparators. A graphical depiction of this study is presented in eFigure 1 in Supplement 1.

### Statistical Analysis

To compare characteristics between the 2 groups and adjust the association on obstetric outcomes, patients’ demographic characteristics, lifestyle factors, and comorbidities before the index date and each pregnancy were identified. Demographic characteristics included age and quintile of practice-level deprivation. Lifestyle factors included body mass index, alcohol intake, and smoking status, and were determined using existing algorithms.^21,22,23^ Alcohol intake was categorized into nondrinker, occasional/light drinker, former drinker, moderate drinker, and heavy drinker based on UK guidance. Comorbidities that might be associated with fertility and pregnancy outcomes were identified according to published criteria.^24^

During the entire follow-up period and each calendar year, fertility rates, defined as the number of pregnancies per 100 patient-years, were calculated. The annual fertility rate in each age category (<20, 20-24, 25-29, 30-34, 35-39, and ≥40 years) was also calculated. A negative binomial model and an offset term of follow-up time was used to identify the association between psoriasis and the fertility rate. The results are presented as rate ratios (RRs) and 95% CIs.

Logistic regression was used to assess the association of birth outcomes and adverse pregnancy outcomes between patients with psoriasis and matched comparators after adjusting for potential confounding factors. The parsimonious models that contained psoriasis and potential confounding factors were presented after eliminating nonsignificant terms based on the likelihood ratio test. The results are presented as adjusted odds ratios (aORs) and 95% CIs. We conducted several sensitivity analyses to assess the robustness of the findings (eMethods in Supplement 1).

The threshold for statistical significance was a 2-sided *P* = .05. All statistical analyses were conducted using Stata, version 16 (StataCorp).

## Results

### Baseline Characteristics of the Study Cohort

Overall, there were 4 224 536 female patients aged 15 to 44 years in the CPRD GOLD during 1998 through 2019, and 63 681 patients with psoriasis and 318 405 comparators were included in this study. The median (IQR) age on the index date was 30 (22-37) years, and the median (IQR) follow-up duration was 4.1 (1.7-7.9) years. There were 3252 patients (5.1%) with moderate to severe psoriasis in the follow-up period, and 561 were assigned as having moderate to severe psoriasis on the index date.

Compared with matched comparators, patients with psoriasis were more likely to be current smokers, overweight, or current alcohol drinkers on the index date. Higher proportions of patients with psoriasis were diagnosed with alcohol use disorders and psychological diseases, such as depression and anxiety; in addition, patients with psoriasis were more often diagnosed with diabetes, hypertension, inflammatory bowel disease, thyroid disorders, inflammatory polyarthropathies, systemic connective tissue disorders, and respiratory diseases, such as asthma and chronic obstructive pulmonary disease (Table 1).

**Table 1.** Baseline Characteristics of Patients With Psoriasis and Matched Comparators Without Psoriasis

Characteristic	No. (%)		*P* value
	Psoriasis (n = 63 681)	Matched comparators (n = 318 405)	
Age, median (IQR), y	30 (22-37)	30 (22-37)	NA^a^
Age category, y			
<20	11 787 (18.5)	58 932 (18.5)	NA^a^
20-24	8955 (14.1)	44 776 (14.1)	
25-29	10 707 (16.8)	53 538 (16.8)	
30-34	11 696 (18.4)	58 483 (18.4)	
35-39	10 938 (17.2)	54 683 (17.2)	
≥40	9598 (15.1)	47 993 (15.1)	
Quintile of index of multiple deprivation			
1 (Least deprived)	10 340 (16.2)	51 700 (16.2)	NA^a^
2	10 711 (16.8)	53 555 (16.8)	
3	11 855 (18.6)	59 275 (18.6)	
4	12 805 (20.1)	64 025 (20.1)	
5 (Most deprived)	17 970 (28.2)	89 850 (28.2)	
Body mass index			
Normal	16 406 (25.8)	79 671 (25.0)	<.001
Overweight	11 185 (17.6)	49 371 (15.5)	
Obesity	11 100 (17.4)	40 734 (12.8)	
Underweight	3748 (5.9)	18 775 (5.9)	
Missing	21 242 (33.4)	129 854 (40.8)	
Smoking status			
Never smoked	22 993 (36.1)	131 301 (41.2)	<.001
Former smoker	9297 (14.6)	39 110 (12.3)	
Current smoker	22 211 (34.9)	89 305 (28.1)	
Missing	9180 (14.4)	58 689 (18.4)	
Alcohol intake			
Nondrinker	5583 (8.8)	28 143 (8.8)	<.001
Occasional/light drinker	8898 (14.0)	43 634 (13.7)	
Former drinker	4196 (6.6)	18 438 (5.8)	
Moderate drinker	19 766 (31.0)	95 464 (30.0)	
Heavy drinker	2974 (4.7)	10 818 (3.4)	
Missing	22 264 (35.0)	121 908 (38.3)	
Psychological disease or substance use issue			
Depression	8460 (13.3)	29 142 (9.2)	<.001
Anxiety^b^	2490 (3.9)	8265 (2.6)	
Psychoactive substance misuse	882 (1.4)	2855 (0.9)	
Alcohol use disorder	578 (0.9)	1910 (0.6)	
Schizophrenia or bipolar disorder	419 (0.7)	1468 (0.5)	
Cardiovascular disease			
Diabetes	717 (1.1)	2701 (0.9)	<.001
Hypertension	1134 (1.8)	4796 (1.5)	<.001
Coronary heart disease	101 (0.2)	377 (0.1)	.009
Gastroenterology disease			
Inflammatory bowel disease	478 (0.8)	1576 (0.5)	<.001
Irritable bowel syndrome	4593 (7.2)	20 637 (6.5)	<.001
Peptic ulcer disease	200 (0.3)	921 (0.3)	.29
Respiratory disease			
Asthma	4873 (7.7)	21 518 (6.8)	<.001
Chronic obstructive pulmonary disease	81 (0.1)	236 (0.1)	
Other conditions			
Rheumatoid arthritis^c^	1591 (2.5)	1850 (0.6)	<.001
Thyroid disorders	1624 (2.6)	6482 (2.0)	<.001
Epilepsy	470 (0.7)	2255 (0.7)	.04
Chronic liver disease	119 (0.2)	564 (0.2)	.60
Hearing loss	2767 (4.4)	13 088 (4.1)	.007
Migraine	252 (0.4)	1105 (0.4)	.06
Systemic lupus erythematosus	84 (0.1)	340 (0.1)	.08

Abbreviation: NA, not applicable.

^a^ These factors were matched during comparators selection.

^b^ Including other neurotic, stress-related, and somatoform disorders.

^c^ Including polyarthritis and other inflammatory systemic connective tissue disorders.

### Trends of Annual Rates of Fertility

Over the study period, patients with psoriasis had higher rates of fertility (RR, 1.30; 95% CI, 1.27-1.33; *P* < .001) compared with age- and practice-matched patients without psoriasis. Following stratification according to the severity of psoriasis, patients with moderate to severe psoriasis had lower rates of fertility (RR, 0.75; 95% CI, 0.69-0.83; *P* < .001).

**Figure 2.**
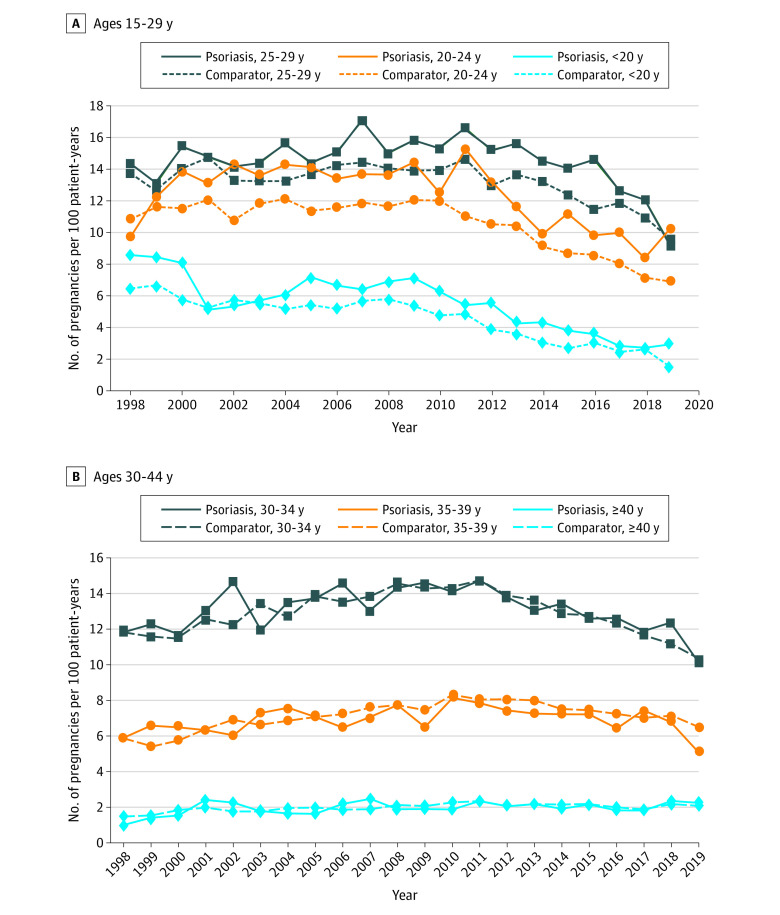
Annual Fertility Rates for Female Patients With Psoriasis and Matched Comparators by Age

The annual fertility rate of patients with psoriasis increased from 8.5 per 100 patient-years in 1998 to 9.8 per 100 patient-years in 2011 and decreased to 6.5 per 100 patient-years in 2019 (eFigure 2 in Supplement 1). Examining the annual fertility rates of patients in different age groups identified a higher annual fertility rate in some calendar years for patients with psoriasis who were 29 years or younger. Compared with matched comparators, patients with psoriasis who were older than 29 years had a similar annual fertility rate (Figure 2).

### Risk of Adverse Birth Outcomes

Overall, 29 657 and 122 115 pregnancies in patients with psoriasis and matched comparators, respectively, between 1998 and 2019 were included. Pregnancies in patients with psoriasis were less likely to end in live birth (OR, 0.91; 95% CI, 0.88-0.93; *P* < .001). In addition, the odds of pregnancy loss were statistically significantly higher in patients with psoriasis (OR, 1.06; 95% CI, 1.03-1.10; *P* < .001), and more than 95% of these (5596 of 5860) were at a gestation period shorter than 91 days. The number needed to harm for pregnancy loss was approximately 107 when comparing patients with psoriasis with age-matched comparators without psoriasis. In addition to psoriasis, patients younger than 20 years (OR, 2.04; 95% CI, 1.94-2.15; *P* < .001) and between the ages of 20 and 24 years (OR, 1.35; 95% CI, 1.31-1.40; *P* < .001) had a higher risk of pregnancy loss compared with patients between the ages of 25 and 34 years. There was no statistically significant difference in stillbirth (OR, 0.96; 95% CI, 0.74-1.26; *P* = .78) and preterm birth (OR, 1.03; 95% CI, 0.96-1.09; *P* = .43) among pregnancies in the 2 groups. The results were consistent after adjusting for demographic characteristics, lifestyle factors, socioeconomic status, and comorbidities (Table 2).

**Table 2.** Birth Outcomes in Patients With Psoriasis and Matched Comparators Without Psoriasis

Outcome	No. (%)		Odds ratio (95% CI)	
	Pregnancies in patients with psoriasis (n = 29 657)	Pregnancies in matched comparators (n = 122 115)	Crude	Adjusted^a^
Live birth	16 078 (54.2)	69 120 (56.6)	0.91 (0.88-0.93)	0.91 (0.88-0.93)
Pregnancy loss	5860 (19.8)	22 998 (18.8)	1.06 (1.03-1.10)	1.05 (1.01-1.08)
Stillbirth	67 (0.2)	286 (0.2)	0.96 (0.74-1.26)	0.96 (0.73-1.25)
Preterm birth^b^	1350 (8.3)	5668 (8.1)	1.03 (0.96-1.09)	1.01 (0.95-1.08)
**Outcome**	**Pregnancies in patients with moderate to severe psoriasis (n = 839)**	**Pregnancies in matched comparators (n = 1588)**	**Crude**	**Adjusted^a^**
Live birth	433 (51.6)	902 (56.8)	0.81 (0.69-0.96)	0.82 (0.70-0.97)
Pregnancy loss	174 (20.7)	293 (18.5)	1.16 (0.94-1.43)	1.18 (0.95-1.46)
Stillbirth^c^	NA	NA	NA	NA
Preterm birth^d^	36 (8.3)	74 (8.2)	1.01 (0.67-1.54)	1.01 (0.67-1.54)

Abbreviation: NA, not available.

^a^ Adjusted by demographic characteristics, lifestyle factors, and comorbidities before the start of pregnancy. Only results from the parsimonious model that contained psoriasis and potential confounding factors were presented after eliminating nonsignificant terms.

^b^ The analysis included only pregnancies with delivery records (16 223 among those with psoriasis and 69 681 among matched comparators).

^c^ Due to the low number of stillbirths, the results were not reported, in line with data governance requirements to minimize potential patient disclosure.

^d^ The analysis included only pregnancies with delivery records (436 among those with moderate to severe psoriasis and 908 among matched comparators).

Pregnancies in patients with moderate to severe psoriasis were less likely to end in live birth compared with matched comparators without psoriasis after adjusting for potential confounding factors (aOR, 0.82; 95% CI, 0.70-0.97; *P* = .02). There were no statistically significant differences in the risks of pregnancy loss, stillbirth, and preterm birth between patients with moderate to severe psoriasis and matched comparators (Table 2). The risks of adverse birth outcomes in patients with mild psoriasis were similar to these results, including among pregnancies in all patients with psoriasis (eTable 2 in Supplement 1).

### Risk of Adverse Pregnancy Outcomes

Overall, 13 247 and 52 773 pregnancies in patients with psoriasis and matched comparators, respectively, were able to be linked to HES records. There was an increased risk of venous thromboembolism in pregnant patients with psoriasis (OR, 1.31; 95% CI, 1.07-1.59; *P* = .008). However, there were no statistically significant differences in the risks of antenatal hemorrhage, preeclampsia, gestational hypertension, gestational diabetes, and cesarean delivery between 2 groups. After adjustment for potential confounding factors, there was no statistically significant difference in the odds of venous thromboembolism (aOR, 1.12; 95% CI, 0.91-1.37; *P* = .28; Table 3). Due to the limited number of adverse pregnancy outcomes in patients with moderate to severe psoriasis and matched comparators, the results are not presented, in line with data governance requirements to minimize potential patient disclosure. The results from the sensitivity analysis were consistent with the primary analysis (eTables 3 and 4 in Supplement 1).

**Table 3.** Adverse Pregnancy Outcomes in Patients With Psoriasis and Matched Comparators Without Psoriasis

Outcome	No. (%)		Odds ratio (95% CI)	
	Pregnancies in patients with psoriasis (n = 13 247)	Pregnancies in matched comparators (n = 52 773)	Crude	Adjusted^a^
Venous thromboembolism	134 (1.0)	410 (0.8)	1.31 (1.07-1.59)	1.12 (0.91-1.37)
Antenatal hemorrhage	1682 (12.7)	6689 (12.7)	1.00 (0.95-1.06)	0.97 (0.92-1.03)
Preeclampsia	187 (1.4)	803 (1.5)	0.93 (0.79-1.09)	0.91 (0.78-1.07)
Gestational hypertension^b^	614 (4.7)	2505 (4.8)	0.97 (0.89-1.07)	0.94 (0.86-1.03)
Gestational diabetes^c^	319 (2.4)	1215 (4.3)	1.05 (0.93-1.19)	0.99 (0.87-1.13)
Cesarean delivery^d^	1200 (18.9)	4734 (18.5)	1.03 (0.96-1.10)	1.02 (0.95-1.10)

^a^ Adjusted by demographic characteristics, lifestyle factors, and comorbidities before the start of pregnancy. Only results from the parsimonious model that contained psoriasis and potential confounding factors were presented after eliminating nonsignificant terms.

^b^ The analysis included only pregnancies in which the patient was not diagnosed with hypertension before the start of pregnancy (13 119 among those with psoriasis and 52 241 among matched comparators).

^c^ The analysis included only pregnancies in which the patient was not diagnosed with diabetes before the start of pregnancy (13 145 among those with psoriasis and 52 447 among matched comparators).

^d^ The analysis included only pregnancies with delivery records and linkage eligibility to Hospital Episode Statistics maternity data (6362 among those with psoriasis and 25 647 among matched comparators).

## Discussion

Higher fertility rates were found in patients with psoriasis, especially among those aged between 15 and 29 years, but lower fertility rates were found in patients who had moderate to severe psoriasis. Overall, the fertility rates of patients with psoriasis followed the national fertility trends.^25^ However, live birth was less likely to occur in patients with psoriasis. In addition, the risk of pregnancy loss was higher when compared with the matched population, and most of these losses occurred in the first trimester. A higher proportion of pregnant patients with psoriasis were diagnosed with venous thromboembolism, but no statistically significant difference was found after adjustment for potential confounding factors.

A US study that used a claims database showed a small increase in fertility rate (RR, 1.1; 95% CI, 1.0-1.2) in women with psoriasis.^26^ The present study found a higher fertility rate (RR, 1.30; 95% CI, 1.27-1.33) when using a pregnancy registry that systematically identified pregnancies.^27^ Underrecording fertility in a claims database would lead to a nondifferential misclassification error.^26,28^ Regarding birth outcomes, Lima et al^29^ included 122 women with psoriasis and 290 comparators in their study and reported a nonsignificant association between psoriasis and pregnancy loss due to the limited sample size. By comparing more than 63 000 patients with psoriasis with 318 000 comparators, the present study found a statistically significant association between psoriasis and pregnancy loss, and the results are similar to a case-control study.^30^ Consistent with studies from the US, Denmark, Sweden, and Taiwan,^29,31,32^ we also showed no difference in preterm birth.

A meta-analysis showed a higher risk of preeclampsia, gestational hypertension, and gestational diabetes in women with psoriasis^15^; this differs from the present findings. However, due to the variation in study design, marked heterogeneity existed even though subgroup analyses were performed. Previous published studies reported no statistically significant increases in the risk of gestational hypertension and preeclampsia in patients with psoriasis, which might be related to the small sample sizes.^16,31^ However, the present study echoes their results when using a much larger UK primary care database. Although cardiovascular diseases were common in patients with psoriasis, we only included female patients of common reproductive ages, and this might explain the nonsignificant association with gestational hypertension and gestational diabetes.

Of particular note, we observed an increased risk of pregnancy loss in patients with psoriasis. The mechanism to link the higher risk of pregnancy loss in patients with psoriasis is not clear, but there might be potential explanations. Psoriasis is characterized by the increased activity of IL-17, IL-23, and tumor necrosis factor α.^33^ Those proinflammatory cytokines may negatively affect the placenta and cause impaired fetal growth.^34,35^

Pregnancy loss has been linked with adverse consequences, such as depression,^36,37^ and the annual national economic cost of pregnancy loss is around £471 million in the UK.^38^ We found that pregnancy loss occurred in about 1 in 5 pregnancies in patients with psoriasis, and approximately 95% of these occurred in the first trimester, which was probably unavoidable due to chromosomal errors. A Danish study indicated a higher risk of ectopic pregnancy in women with psoriasis,^30^ which often complicates pregnancies in the first trimester and might explain the higher risk of loss in the present study. Due to the small effect size of pregnancy loss (19.8% vs 18.8%), it is unclear if better management of psoriasis and close monitoring could balance maternal benefit and risk. There is a need for studies exploring the mechanism of pregnancy loss in patients with psoriasis. In addition, general practitioners in primary care, where the diagnosis of psoriasis may be missed and hence the treatment may be delayed, should be aware of the increased risk of early pregnancy loss, especially for younger patients with psoriasis.^39^

This study used a large EHR database that is representative in terms of age and gender distribution, and hence the results could be generalized to the population.^40,41^ To the best of our knowledge, this is one of the largest studies investigating fertility and obstetric outcomes in psoriasis.^14,17,32^ Linkage with pregnancy registry and HES data provides more accurate records regarding timing and outcomes of pregnancies, minimizing misclassification of study outcomes.^18^ Compared with administrative birth records, which include only pregnancies that end in live births or stillbirths, the use of a pregnancy registry enables the risk of pregnancy loss to be quantified.

### Limitations

There are some limitations. Due to the lack of direct measures of psoriasis severity, we used surrogates for moderate to severe psoriasis, which are commonly used in published studies using EHR databases.^31,32^ Although we applied a large EHR database, due to the lower fertility rate, there were a limited number of adverse pregnancy outcomes in patients with moderate to severe psoriasis. In addition, we could only adjust the influence of ethnicity on adverse pregnancy outcomes that were identified in patients with linkage eligibility to HES records because only a small proportion of all patients in the CPRD GOLD have ethnicity recorded.^42^ Although the majority of patients report their pregnancies to primary care in the UK,^43^ for patients who are not aware of the pregnancy or who did not contact their general practitioner and experienced a miscarriage, they will not be included in the pregnancy register.

## Conclusions

In this cohort study, patients with psoriasis had higher rates of fertility when compared with those without psoriasis, but lower fertility rates were found when patients developed moderate to severe psoriasis. Of particular note, pregnancy loss was more common in patients with psoriasis. To avoid miscarriage and its adverse consequences, further studies should evaluate the effects of better management of psoriasis and close monitoring during pregnancy on pregnancy loss. In particular, patients with psoriasis were more likely to have comorbidities that may be related to poor pregnancy outcomes, and hence increased emphasis of managing comorbidities as part of the routine management plan is also warranted.

## Notes

Supplement 1. **eTable 1.** Definitions of adverse birth outcomes and pregnancy outcomes and the data sources **eTable 2.** Birth outcomes in women with or without mild psoriasis **eTable 3.** The results of sensitivity analysis on fertility and birth outcomes **eTable 4.** The results of sensitivity analysis on pregnancy outcomes **eFigure 1.** Graphical depiction of study design and follow-up **eFigure 2.** Annual rate of fertility of women with psoriasis when compared to matched comparator **eMethods.** Explanation of sensitivity analysis

Supplement 2. Data Sharing Statement
