# Supplementary material for: Fertility Trends and Adverse Pregnancy Outcomes in Female Patients With Psoriasis in the UK
Source: JAMA Dermatol. 2023 Jun 7;159(7):736–44. doi: 10.1001/jamadermatol.2023.1400 (PMC10248813; doi:10.1001/jamadermatol.2023.1400)
Supplement: Supplement 1. — eTable 1. Definitions of adverse birth outcomes and pregnancy outcomes and the data sources eTable 2. Birth outcomes in women with or without mild psoriasis eTable 3. The results of sensitivity analysis on fertility and birth outcomes eTable 4. The results of sensitivity analysis on pregnancy outcomes eFigure 1. Graphical depiction of study design and follow-up eFigure 2. Annual rate of fertility of women with psoriasis when compared to matched comparator eMethods. Explanation of sensitivity analysis [file jamadermatol-e231400-s001.pdf]

Supplemental Online Content

Chen TC, Iskandar IYK, Parisi R, et al; for the Global Psoriasis Atlas. Fertility trends and adverse pregnancy outcomes in female patients with psoriasis in the UK. *JAMA Dermatol*. Published online June 7, 2023. doi:10.1001/jamadermatol.2023.1400

eTable 1. Definitions of adverse birth outcomes and pregnancy outcomes and the data sources
eTable 2. Birth outcomes in women with or without mild psoriasis
eTable 3. The results of sensitivity analysis on fertility and birth outcomes
eTable 4. The results of sensitivity analysis on pregnancy outcomes
eFigure 1. Graphical depiction of study design and follow-up
eFigure 2. Annual rate of fertility of women with psoriasis when compared to matched comparator
eMethods. Explanation of sensitivity analysis

This supplemental material has been provided by the authors to give readers additional information about their work.

© 2023 Chen TC et al. *JAMA Dermatology.*

eTable 1. Definitions of adverse birth outcomes and pregnancy outcomes and the data sources

Outcome	Data source	Definition
Pregnancy loss	Pregnancy register	Pregnancies with the outcome recorded as miscarriage, ectopic, molar, blighted ovum and any unspecified loss.
Live-birth	Pregnancy register	Any pregnancy episode with an outcome recorded as:<br>1. live-birth,<br>2. delivery based on a third trimester pregnancy record, or<br>3. delivery based on the late pregnancy record
Stillbirth	Pregnancy register	Pregnancies with the outcome recorded as still-birth.
Pre-term birth	Pregnancy register	Delivered pregnancy episodes with less than 259 days (37 weeks) of gestation period
Venous thromboembolism	HES and CPRD	Any pregnancy episode with ICD codes in HES or Read codes in CPRD from the start of pregnancy to 6 weeks after delivery
Antenatal haemorrhage	HES and CPRD	Any pregnancy episode with ICD codes in HES or Read codes in CPRD from the start of pregnancy to deliver
Preeclampsia	HES and CPRD	Any pregnancy episode with ICD codes in HES or Read codes in CPRD from the 20th week of pregnancy to 4 weeks after deliver
Gestational hypertension	HES and CPRD	Any pregnancy episode with ICD codes in HES or Read codes in CPRD from the 20th week of pregnancy to 4 weeks after deliver
Gestational Diabetes	HES and CPRD	Any pregnancy episode with ICD codes in HES or Read codes in CPRD from the 16th week of pregnancy to 4 weeks after deliver
Caesarean	HES	Delivered pregnancy episodes with method of deliver recorded as caesarean

© 2023 Chen TC et al. *JAMA Dermatology.*

eTable 2. Birth outcomes in women with or without mild psoriasis

	Pregnancies in women with mild psoriasis (n=28,818)	Pregnancies in matched comparators (n=120,527)		
	**Number (absolute risk)**	**Number (absolute risk)**	**Crude OR (95%CI)**	**Adjusted OR [a] (95%CI)**
Live birth	15645 (54.29%)	68218 (56.60%)	0.91 (0.89 to 0.93)	0.91 (0.88 to 0.93)
Pregnancy loss	5686 (19.73%)	22705 (18.84%)	1.06 (1.03 to 1.10)	1.04 (1.01 to 1.08)
Still-birth	66 (0.23%)	282 (0.23%)	0.98 (0.75 to 1.28)	0.97 (0.74 to 1.27)
Pre-term birth [b]	1314 (8.32%)	5594 (8.13%)	1.03 (0.96 to 1.09)	1.01 (0.95 to 1.08)

(Note) [a] The odds ratio (OR) was adjusted by demographic characteristics, lifestyle factors and comorbidities before the start of pregnancy and only the parsimonious model that contained psoriasis and potential confounding factors were presented after eliminating non-significant terms. [b] The analysis included only pregnancies with delivery records (psoriasis:15787, matched comparators: 68773).

© 2023 Chen TC et al. *JAMA Dermatology.*

eTable 3. The results of sensitivity analysis on fertility and birth outcomes

	Fertility rate ratios (95%CI)	Live-birth odds ratio (95%CI)	Pregnancy loss odds ratio (95%CI)	Still-birth odds ratio (95%CI)	Pre-term birth odds ratio (95%CI)
Original results	1.30 (1.27 to 1.33)	0.91 (0.88 to 0.93)	1.05 (1.01 to 1.08)	0.96 (0.73 to 1.25)	1.01 (0.95 to 1.08)
Excluding women diagnosed with infertility before the index date	1.32 (1.29 to 1.35)	0.91 (0.88 to 0.93)	1.04 (1.01 to 1.08)	0.96 (0.73 to 1.26)	1.01 (0.95 to 1.08)
Excluding women prescribed with intrauterine contraceptive devices within 3 years before the index date	1.29 (1.27 to 1.32)	0.91 (0.88 to 0.93)	1.05 (1.01 to 1.08)	0.93 (0.71 to 1.23)	1.01 (0.95 to 1.08)
Excluding women prescribed with contraception within 3 months before the index date	1.30 (1.27 to 1.33)	0.90 (0.87 to 0.93)	1.07 (1.03 to 1.11)	0.97 (0.73 to 1.31)	1.01 (0.94 to 1.09)
Excluding women diagnosed with psoriatic arthritis before the index date	1.30 (1.28 to 1.33)	0.90 (0.88 to 0.93)	1.05 (1.01 to 1.08)	0.97 (0.75 to 1.27)	1.02 (0.95 to 1.08)
Pregnancy episodes with unknown outcomes were included as live-births	NA	0.96 (0.93 to 0.99)	NA	NA	NA
Pregnancy episodes with unknown outcomes were included as pregnancy loss	NA	NA	1.10 (1.07 to 1.13)	NA	NA

Note: NA: not applicable. 95%CI: 95% confidence interval

© 2023 Chen TC et al. *JAMA Dermatology.*

eTable 4. The results of sensitivity analysis on pregnancy outcomes

	**Venous thromboembolism**	**Antenatal haemorrhage**	**Preeclampsia**	**Gestational hypertension**	**Gestational diabetes**	**Caesarean**
Original results	1.12 (0.91 to 1.37)	0.97 (0.92 to 1.03)	0.91 (0.78 to 1.07)	0.94 (0.86 to 1.03)	0.99 (0.87 to 1.13)	1.02 (0.95 to 1.10)
Excluding women diagnosed with infertility before the index date	1.11 (0.90 to 1.36)	0.97 (0.91 to 1.03)	0.91 (0.77 to 1.07)	0.93 (0.85 to 1.03)	0.97 (0.86 to 1.13)	1.04 (0.97 to 1.12)
Excluding women prescribed with intrauterine contraceptive devices within 3 years before the index date	1.15 (0.94 to 1.41)	0.98 (0.92 to 1.04)	0.92 (0.78 to 1.08)	0.96 (0.87 to 1.05)	0.99 (0.87 to 1.12)	1.02 (0.95 to 1.10)
Excluding women prescribed with contraception within 3 months before the index date	1.08 (0.86 to 1.36)	0.97 (0.91 to 1.04)	0.98 (0.81 to 1.18)	0.95 (0.85 to 1.05)	0.98 (0.85 to 1.14)	1.02 (0.94 to 1.11)
Excluding women diagnosed with psoriatic arthritis before the index date	1.11 (0.90 to 1.36)	0.97 (0.92 to 1.03)	0.92 (0.78 to 1.08)	0.94 (0.86 to 1.04)	0.99 (0.87 to 1.13)	1.02 (0.95 to 1.10)

Note: the results are presented as odds ratio (95% confidence interval)

© 2023 Chen TC et al. *JAMA Dermatology.*

eFigure 1. Graphical depiction of study design and follow-up

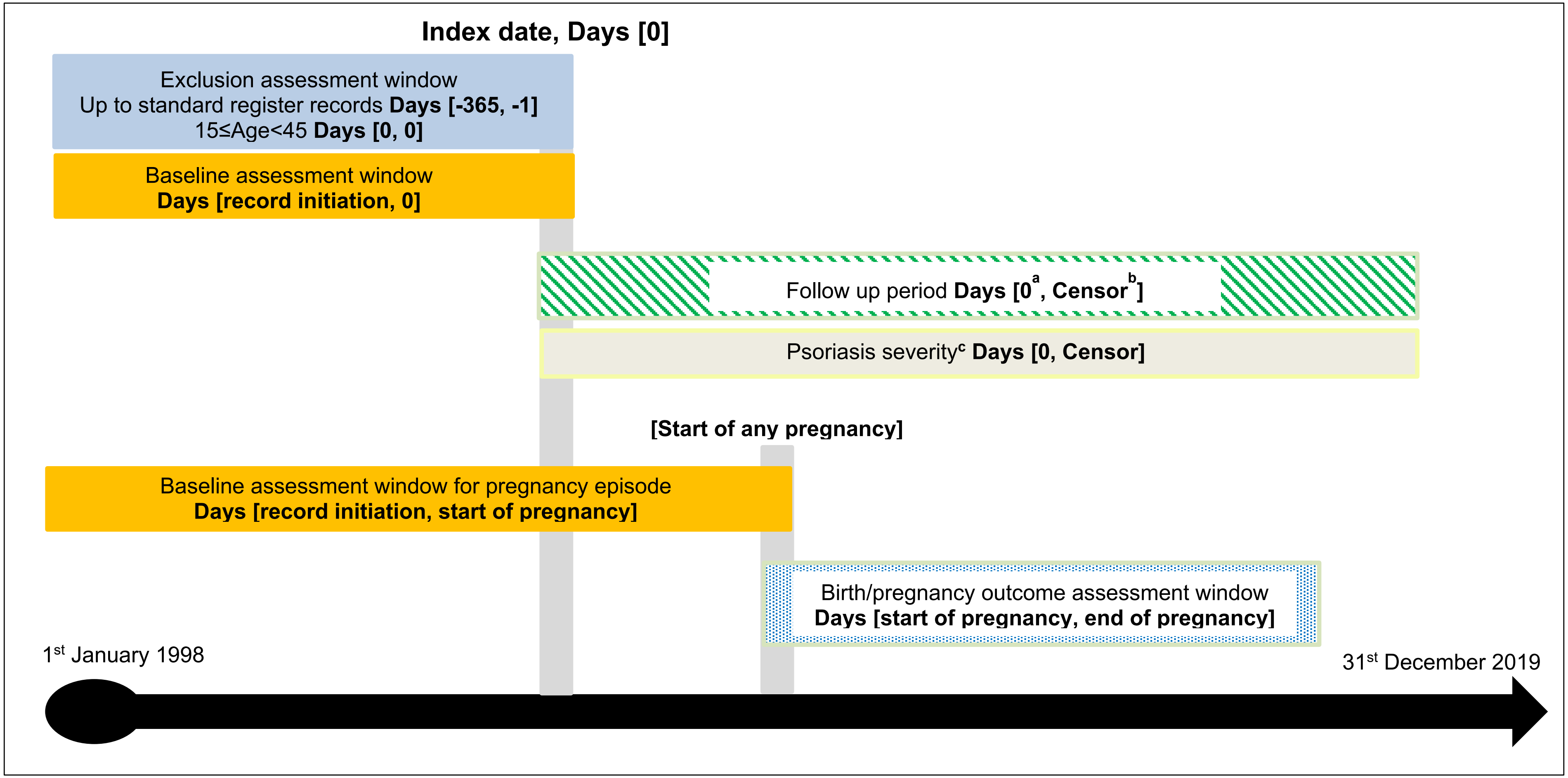

Note: [a] The latest date of age 15, 1st January 1998 or the diagnosis of psoriasis in the study window; [b] First occur of age 45, 31st December 2019, death, transfer out or last data collect; [c] Women with psoriasis were categorised as moderate-to-severe psoriasis when systemic treatments, phototherapy or biologic therapies were prescribed and hence the severity of psoriasis was a time-dependent variable.

© 2023 Chen TC et al. *JAMA Dermatology.*

eFigure 2. Annual rate of fertility of women with psoriasis when compared to matched comparator

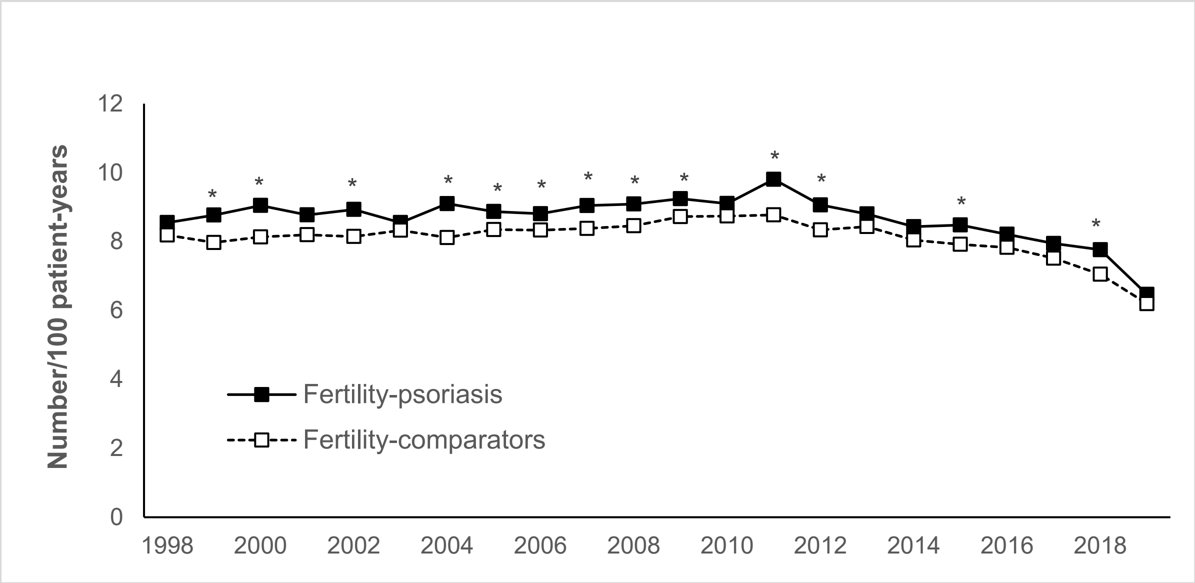

© 2023 Chen TC et al. *JAMA Dermatology.*

eMethods. Explanation of sensitivity analysis

We conducted several sensitivity analyses to assess the robustness of the findings. To identify potential information bias, women who were: (1) diagnosed with infertility before the index date; (2) prescribed with intrauterine contraceptive devices within 3 years before the index date; or (3) prescribed contraception within 3 months before the index date were excluded in each sensitivity analysis. In addition, as psoriatic arthritis is associated with cardiovascular diseases which could impact on pregnancy outcomes, patients diagnosed with psoriatic arthritis before the index date were excluded in one of the sensitivity analysis. Moreover, to differentiate the influence of potential underestimate of live-births, pregnancy episodes with unknown outcomes were included as live-births or pregnancy loss in the sensitivity analysis.

© 2023 Chen TC et al. *JAMA Dermatology.*
