# Supplementary material for: Fertility Trends and Adverse Pregnancy Outcomes in Female Patients With Psoriasis in the UK
Source: JAMA Dermatol. 2023 Jun 7;159(7):736–44. doi: 10.1001/jamadermatol.2023.1400 (PMC10248813; doi:10.1001/jamadermatol.2023.1400)
Supplement: Supplement 2. — Data Sharing Statement [file jamadermatol-e231400-s002.pdf]

Data Sharing Statement

Chen. Fertility Trends and Adverse Pregnancy Outcomes in Female Patients With Psoriasis in the UK. *JAMA Dermatol*. Published June 07, 2023. doi:10.1001/jamadermatol.2023.1400

Data
Data available: No

Additional Information
Explanation for why data not available: Electronic health records are, by definition, considered sensitive data in the UK by the Data Protection Act and cannot be shared via public deposition because of information governance restriction in place to protect patient confidentiality. Access to data is available once approval has been obtained through the individual constituent entities controlling access to the data. The primary care data and pregnancy register can be requested via application to the Clinical Practice Research Datalink.
